# Functional Role of Dimerization of Human Peptidylarginine Deiminase 4 (PAD4)

**DOI:** 10.1371/journal.pone.0021314

**Published:** 2011-06-22

**Authors:** Yi-Liang Liu, Yu-Hsiu Chiang, Guang-Yaw Liu, Hui-Chih Hung

**Affiliations:** 1 Department of Life Sciences and Institute of Genomics and Bioinformatics, National Chung-Hsing University, Taichung, Taiwan; 2 Institute of Microbiology & Immunology, Chung Shan Medical University, and Division of Allergy, Immunology, and Rheumatology, Chung Shan Medical University Hospital, Taichung, Taiwan; University of Leuven, Rega Institute, Belgium

## Abstract

Peptidylarginine deiminase 4 (PAD4) is a homodimeric enzyme that catalyzes Ca^2+^-dependent protein citrullination, which results in the conversion of arginine to citrulline. This paper demonstrates the functional role of dimerization in the regulation of PAD4 activity. To address this question, we created a series of dimer interface mutants of PAD4. The residues Arg8, Tyr237, Asp273, Glu281, Tyr435, Arg544 and Asp547, which are located at the dimer interface, were mutated to disturb the dimer organization of PAD4. Sedimentation velocity experiments were performed to investigate the changes in the quaternary structures and the dissociation constants (*K*_d_) between wild-type and mutant PAD4 monomers and dimers. The kinetic data indicated that disrupting the dimer interface of the enzyme decreases its enzymatic activity and calcium-binding cooperativity. The *K*_d_ values of some PAD4 mutants were much higher than that of the wild-type (WT) protein (0.45 µM) and were concomitant with lower *k*_cat_ values than that of WT (13.4 s^−1^). The *K*_d_ values of the monomeric PAD4 mutants ranged from 16.8 to 45.6 µM, and the *k*_cat_ values of the monomeric mutants ranged from 3.3 to 7.3 s^−1^. The *k*_cat_ values of these interface mutants decreased as the *K*_d_ values increased, which suggests that the dissociation of dimers to monomers considerably influences the activity of the enzyme. Although dissociation of the enzyme reduces the activity of the enzyme, monomeric PAD4 is still active but does not display cooperative calcium binding. The ionic interaction between Arg8 and Asp547 and the Tyr435-mediated hydrophobic interaction are determinants of PAD4 dimer formation.

## Introduction

Peptidylarginine deiminase (PAD; protein-arginine deiminase; EC 3.5.3.15) is a Ca^2+^-dependent enzyme that catalyzes protein citrullination (deimination) in the presence of Ca^2+^. Protein citrullination, which is catalyzed by the PAD enzyme, is a post-translational modification process that converts arginine to citrulline. The change from a positively charged arginine to a neutral citrulline is thought to induce protein unfolding [1]–[3]. PADs play essential roles in epithelial terminal differentiation [4], nervous growth [5], [6], embryonic development [7], apoptosis [8], [9], and transcriptional regulation of gene expression [10], [11]. Also, some human diseases, such as psoriasis, multiple sclerosis, rheumatoid arthritis Alzheimer's disease, and various types of cancers are associated with the PAD enzymes and their citrullinated targets [12]–[16].

Human peptidylarginine deiminase 4 (PAD4) is one of the PAD isoforms. To date, five isoforms of human PADs, PAD1–4 (PAD5 is the same as PAD4) and PAD6, have been identified, and they display tissue-specific expression patterns. PAD1 is found in the skin epidermis and citrullinates keratins and filaggrins. PAD2 is found in the brain and muscle tissues and citrullinates myelin basic proteins. PAD3 is found in hair follicles and citrullinates trichohyalin. PAD4 is found in granulocytes, monocytes and macrophages and citrullinates histones (H2A, H3 and H4) and nucleophosmin/B23. Finally, PAD6 has been identified in embryonic stem cells and oocytes [3], [17]–[21]. In addition to those listed above, the proteins that have been identified as PAD substrates comprise vimentin [22], fibrin [12], fibrinogen [23], alpha-enolase and collagen type I [13]–[16]. Although some protein substrates of PADs have been identified, the protein substrate specificity of PADs remains unknown. The sequence identities among the five PAD isoforms are approximately 50–55%. The catalytic and calcium binding residues are almost all conserved. PAD4 is the only isoform that is located in the nucleus because PAD4 contains a nuclear localization signal (NLS). Nuclear proteins, histones and nucleophosmin/B23, which are citrullinated by PAD4, may be associated with apoptosis [24]. Some studies have shown that citrullination correlates with specific biological events, such as inflammation, apoptosis, trauma, aging and histone-related gene expression and regulation [10], [11], [25], [26]; however, little is known about the physiological roles of PAD4 and other PAD isoforms *in vivo*.

Rheumatoid arthritis (RA) is a chronic, autoimmune disease that is thought to be associated with PAD4 [27]. This disease produces pain, swelling, and stiffness of the synovial joints, joint inflammation and damage to bone and cartilage. Large numbers of autoantibodies against citrullinated proteins are often detected in the blood of patients with RA [28], including factors that are strongly associated with RA, indicating that the citrullinated proteins in RA patients are major autoantigenic epitopes of anti-citrullinated protein antibodies. A number of antibodies in sera of RA patients, such as anti-perinuclear factor, anti-keratin antibody, anti-Sa antibody (Sa antigen has been suggested to be identical to citrullinated vimentin), anti-cyclic citrullinated peptide (CCP) antibodies and anti-citrullinated fibrinogen (CitFib) antibody, are produced early in the progression of the disease and are more specific for RA than rheumatoid factor [14], [15], [29], [30]. As a result, antibodies against these proteins can be used as diagnostic markers for RA. A genome-wide, single-nucleotide polymorphism (SNP)-based analysis of RA patients identified a specific haplotype of the PAD4 gene, which suggests that this enzyme seems to increase the susceptibility for RA in Japanese and Korean populations [27], [31], [32]. These SNPs in the PAD4 gene are thought to make the PAD4 mRNA more stable [27]. In addition, the PAD4 SNPs increase the PAD4 enzyme activity to enhance apoptosis through the mitochondrial pathway [33]. Furthermore, PAD inhibitors are drug development targets. The Cl- and F-amidine inhibitors are synthesized to effectively inhibit PAD1, 3 and 4 activity [34]–[36].

Overexpression of PAD4 induces apoptosis in hematopoietic cells. PAD4 also mediates cell growth arrest and apoptosis, both of which are associated with expression of the tumor suppressor p53 [37]. PAD4 is involved in the repression of p53 target gene expression which interacts with the C-terminus of p53 and further regulates p53 target genes [26], [38]. Since PAD4 functions as a p53 corepressor, inhibitors of this enzyme are considered potential treatments for cancer therapy. The synthetic Cl- and F-amidine inhibitors have also been used to examine their inhibitory effects on PAD4 activity to evaluate cancer cell survival rates [34], [36], [38].

A recent report has shown that PAD4 is autocitrullinated *in vitro* and *in vivo*, and this modification inactivates enzyme function and augments its recognition by human autoantibodies [39]. As a result, antibodies against these proteins can be used as diagnostic markers for RA. In contrast, Thompson's group reports that autocitrullination of PAD4 and PAD4 SNPs does not alter the enzyme activities, substrate specificity and calcium dependence of the enzyme but alters the ability to bind the PAD4-interacting proteins such as histone deacetylase 1, citrullinated histone H3, and protein methyltransferase 1 [40].

**Figure 1:**
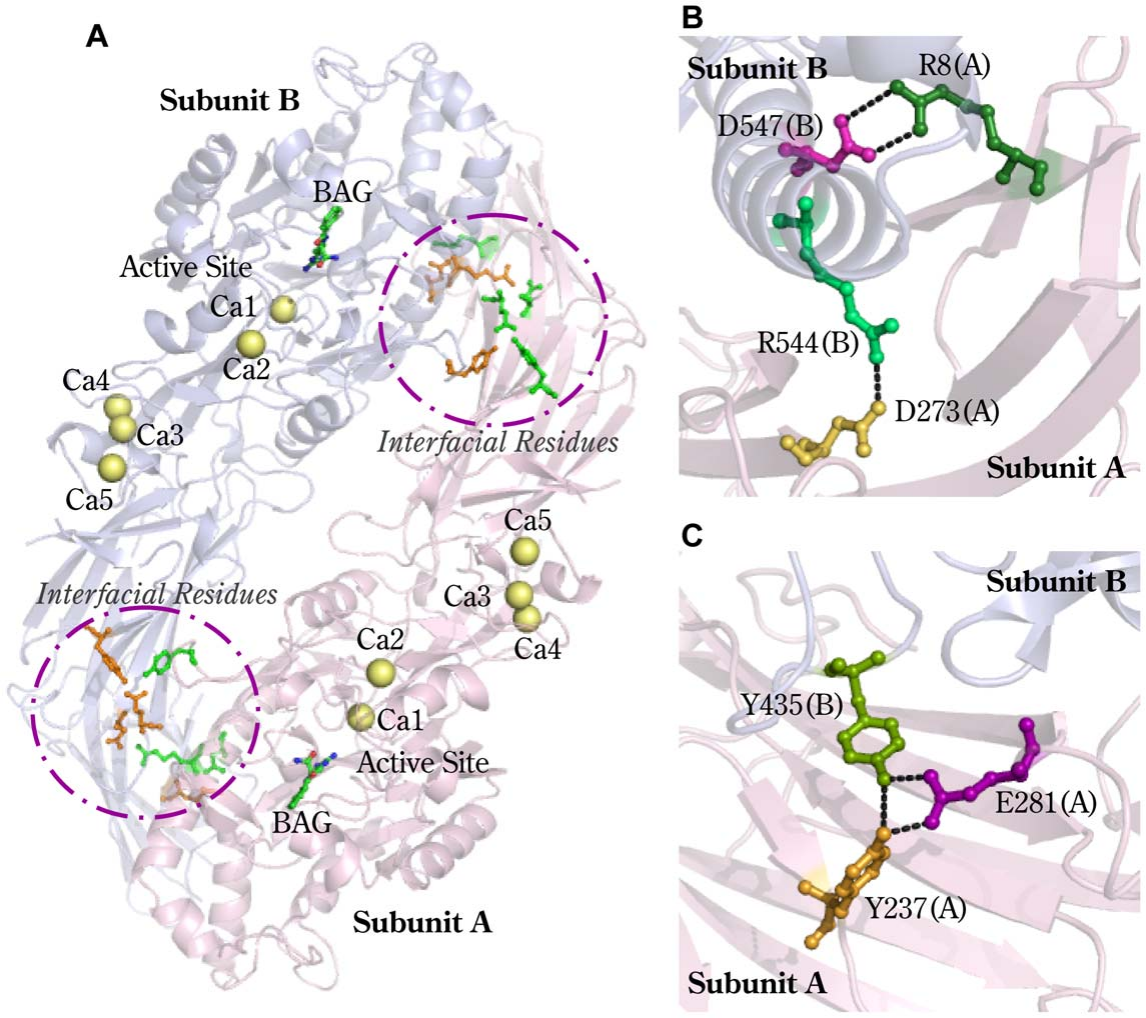
Homodimeric PAD4 and residues located at the dimer interface. Homodimer of human PAD4 (PDB code: 1WDA, **Panel A**). The active site and calcium-binding site are indicated. Five calcium ions (Ca1–Ca5) are indicated as yellow balls. The substrate analog, benzoyl-L-arginine amide (BAG), is shown as a stick model. Panels B and C display the amino acid residues in the dimer interface of PAD4, which are represented by ball-and-stick models. **Panel B**: Arg8(A) is ion-paired with Asp547(B), and Asp273(A) is ion-paired with Arg544(B). **Panel C**: The hydrogen-bonding network formed by Tyr435(B), Tyr237(A) and Glu281(A). The figures were generated with PYMOL (DeLano Scientific LLC, San Carlos, CA, USA).

Multiple X-ray structures of PAD4 in complex with ligands and calcium ions have been solved and are available in the Protein Data Bank [41], [42]. PAD4 is a homodimeric enzyme with an elongated rubber boot structure (Figure 1A). Each monomer contains a separate active site with two calcium ions (Ca1, Ca2) in the C-terminal domain and a distinct binding region with three additional calcium ions (Ca3, Ca4, and Ca5) situated at the N-terminal domain (Figure 1A). Calcium-free, calcium-bound and substrate-bound PAD4 structures indicate that binding of Ca^2+^ to the acidic concave surface of the enzyme induces a conformational change that subsequently creates the active site cleft [41].

The overall quaternary structure of PAD4 shows that PAD4 is a dimer of two monomers that make head-to-tail contact (Figure 1A). Although the dimeric form seems to be the functional unit for PAD4, it is still unclear whether the dimeric form is unique to PAD4 or common to all PADs. Furthermore, the relationship between PAD4 enzyme activity and dimer formation is also unclear. To address these questions, we created a series of PAD4 mutants of residues Arg8, Tyr237, Glu281, Tyr435, and Asp547, which are located on the dimer interface (Figures 1B and 1C). The subunit-subunit interactions at the dimer interface were disrupted by site-directed mutagenesis, which resulted in the dissociation of the dimers into monomers. The quaternary structures of these dimer interface mutants were analyzed by analytical ultracentrifugation. Our data indicate that the PAD4 monomer is less active than the dimer, which suggests that dimerization is required for the full enzyme activity of PAD4.

## Results

The amino acid residues at the dimer interface, including Arg8, Tyr237, Asp273, Glu281, Tyr435, Arg544 and Asp547 (Figure 1), may be involved in subunit-subunit interactions. The following mutants were constructed: R8A, R8E, R8H, R8K, R8L and R8Q; D547A, D547E and D547N; Y237A; D273A; E281A; R544A; and Y435A and Y435N.

### Quaternary Structures of Human WT and Mutant PAD4s

**Figure 2:**
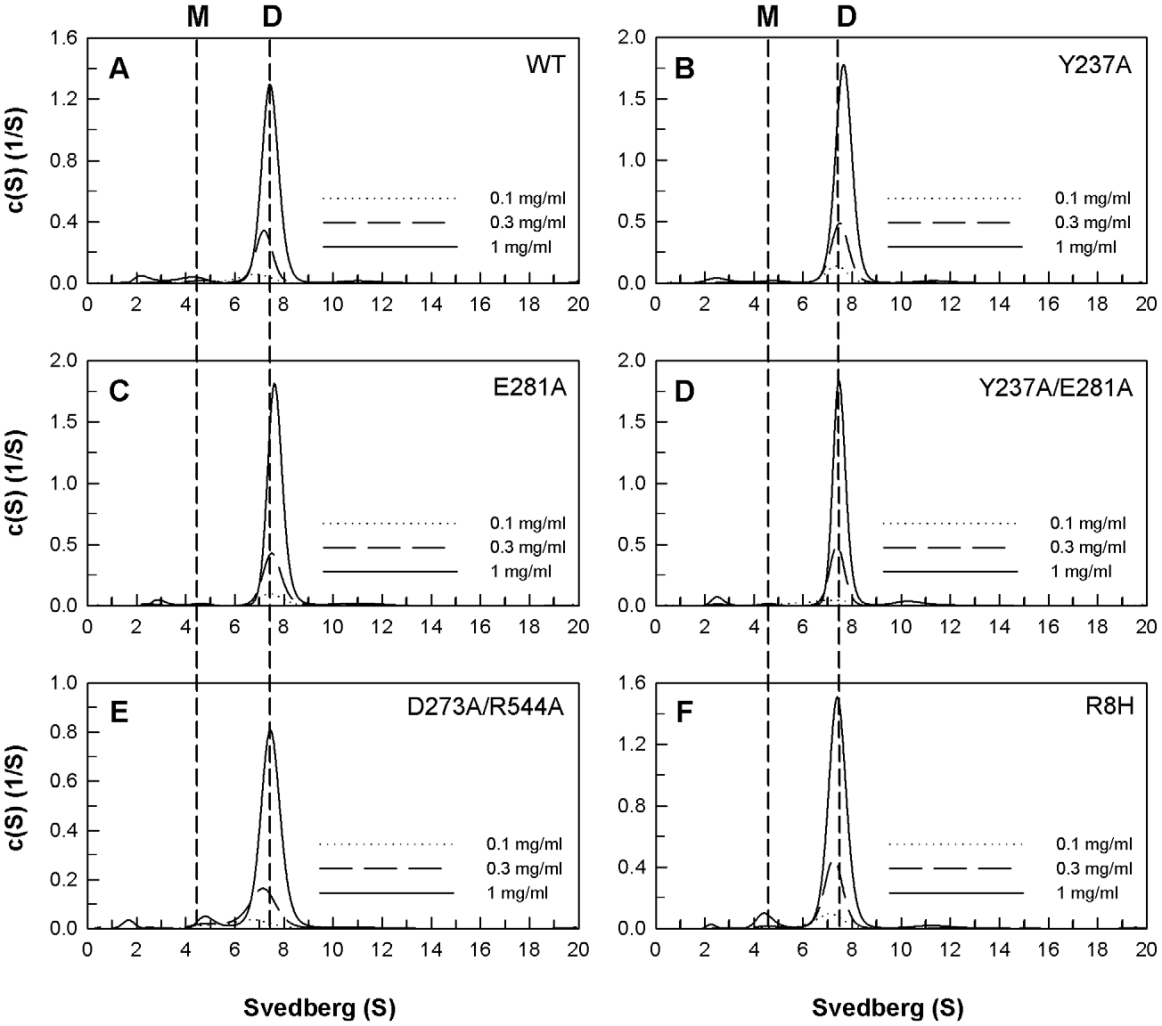
Continuous sedimentation coefficient distributions of the dimeric PAD4 WT and interface mutants. The enzyme concentrations used in the experiments were 0.1, 0.3 and 1 mg/mL in 50 mM Tris-HCl and 250 mM NaCl (pH 7.6) at 20°C. (**A**) WT; (**B**) Y237A mutant; (**C**) E281A mutant; (**D**) Y237A/E281A double mutant; (**E**) D273A/R544A double mutant; (**F**) R8H mutant.

**Figure 3:**
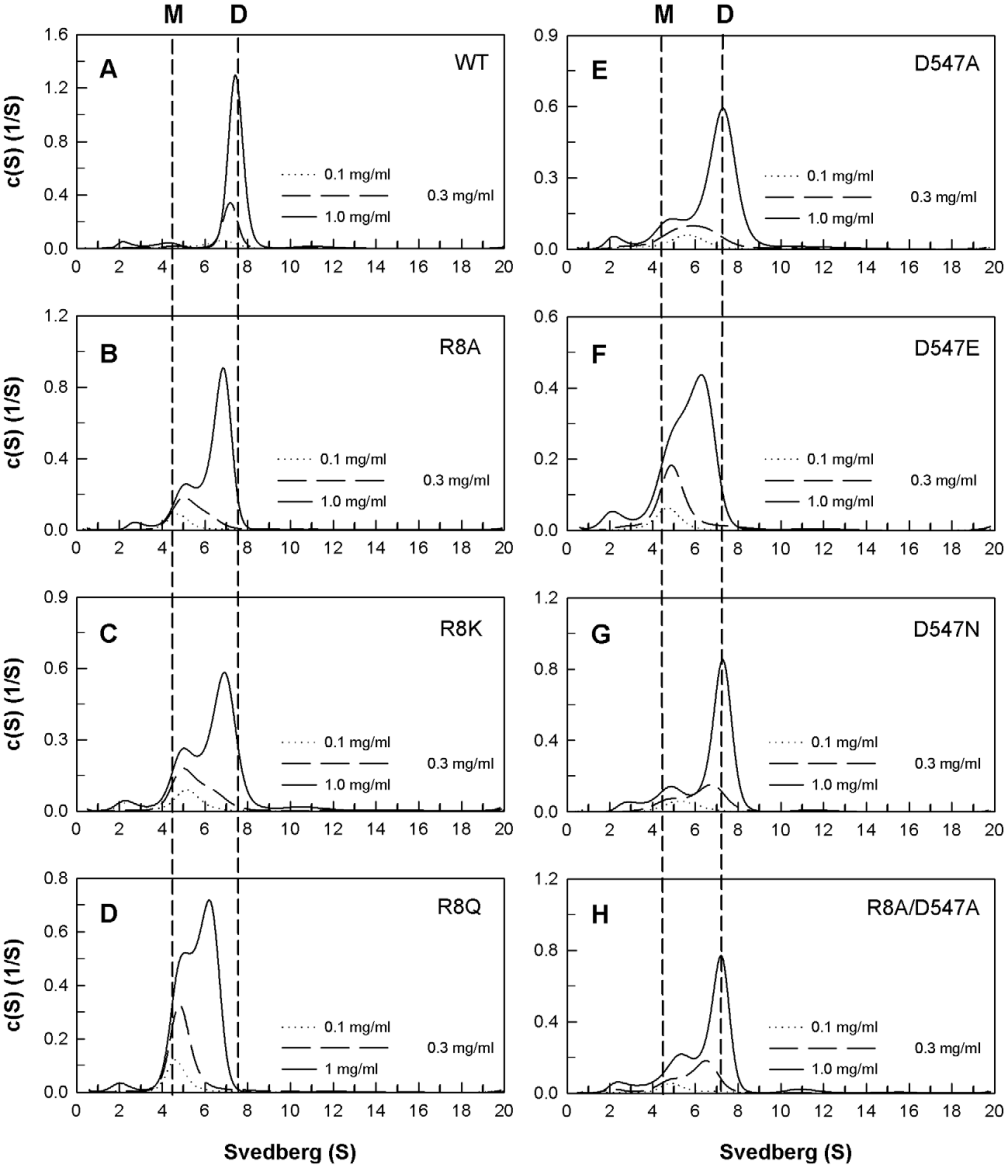
Continuous sedimentation coefficient distributions of the PAD4 interface mutants in monomer-dimer equilibriums. The enzyme concentrations used in the experiments were 0.1, 0.3 and 1 mg/mL in 50 mM Tris-HCl and 250 mM NaCl (pH 7.6) at 20°C. (**A**) WT; (**B**) R8A mutant; (**C**) R8K mutant; (**D**) R8Q mutant; (**E**) D547A mutant; (**F**) D547E mutant; (**G**) D547N mutant; (**H**) R8A/D547A double mutant.

**Figure 4:**
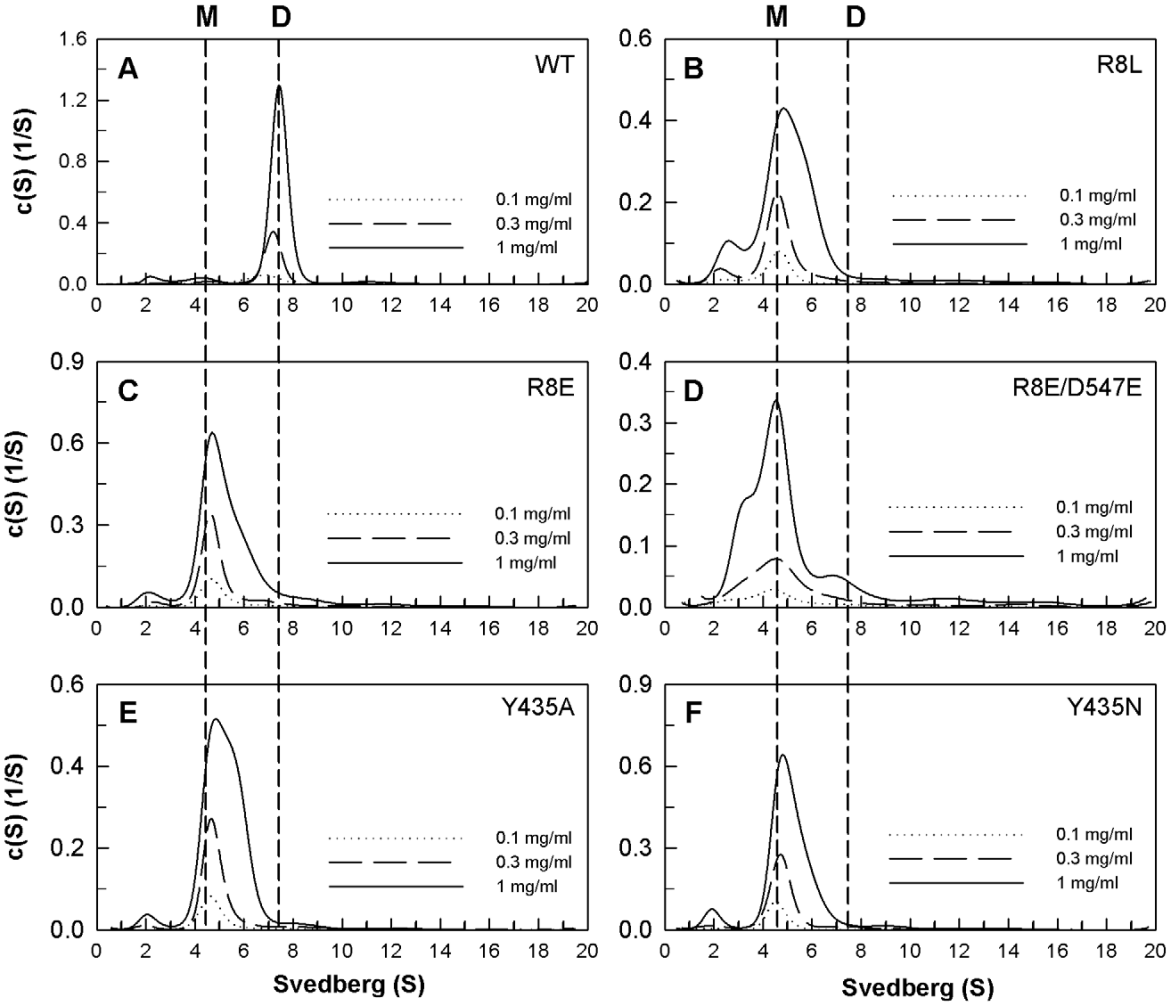
Continuous sedimentation coefficient distributions of the monomeric PAD4 interface mutants. The enzyme concentrations used in the experiments were 0.1, 0.3 and 1 mg/mL in 50 mM Tris-HCl and 250 mM NaCl (pH 7.6) at 20°C. (**A**) WT; (**B**) R8L mutant; (**C**) R8E mutant; (**D**) R8E/D547E double mutant; (**E**) Y435A mutant; (**F**) Y435N mutant.

The quaternary structures of the WT and mutant PAD4 proteins were examined by analytical ultracentrifugation (Figures 2, 3 and 4), and the dissociation constants (*K*_d_) between the dimers and monomers were determined (Table 1). Differences in the size distributions among WT and mutant enzymes were analyzed by sedimentation velocity experiments. The WT enzyme principally exists as a dimer in solution, with a *K*_d_ value of 0.45 µM (Figure 2A). At the dimer interface, Arg8 of subunit A is ion-paired with Asp547 of subunit B, and Asp273 of subunit A is ion-paired with Arg544 of subunit B (Figure 1B). In addition, Tyr237 and Glu281 of subunit A form a hydrogen-bonding network with Tyr435 of subunit B (Figure 1C). The single mutants, Y237A and E281A, existed as dimers, with *K*_d_ values of 0.29 µM and 0.1 µM, respectively (Figure 2, B and C). The double mutants Y237A/E281A and D273A/R544A also dimerized and had *K*_d_ values of 0.1 µM and 0.68 µM, respectively (Figure 2, D and E), which suggests that the salt bridge between Asp273 and Arg544 and the Tyr237 and Glu281-mediated hydrogen-bonding network are insignificant for PAD4 dimer formation.

**Table 1:** Kinetic Parameters of the Human WT and Mutant PAD4s.

PAD4	*K*_m,BAEE_ a (mM)	*K*_0.5,Ca_ b(mM)	*k*_cat_ c(s^−1^)	*h* d	Quaternary Structure^e^	*K*_d_ f(µM)	*K*_d,Ca_ f(µM)
WT	0.31±0.03	0.71±0.02	13.4±0.19	2.30±0.13	D	0.45±0.003	0.51±0.004
R8A	0.47±0.03	0.71±0.04	12.2±0.24	1.50±0.12	M/D	9.3±0.08	ND^g^
R8E	1.06±0.08	1.65±0.41	7.3±0.71	1.01±0.12	M	45.6±0.37	22.3±0.12
R8H	0.47±0.06	0.58±0.04	13.3±0.32	1.71±0.17	D	0.47±0.005	ND^g^
R8K	0.50±0.08	0.82±0.04	10.8±0.19	1.54±0.10	M/D	10.2±0.11	7.8±0.08
R8L	1.36±0.17	1.72±0.52	5.3±0.59	1.06±0.18	M	16.8±0.14	ND^g^
R8Q	0.60±0.07	1.07±0.20	12.2±0.86	1.35±0.29	M/D	15.7±0.14	ND^g^
D547A	0.36±0.05	0.77±0.09	11.6±0.48	1.26±0.16	M/D	6.4±0.07	ND^g^
D547E	0.72±0.10	1.24±0.13	8.9±0.35	1.26±0.11	M/D	11.2±0.11	ND^g^
D547N	0.86±0.10	1.02±0.05	12.2±0.25	1.81±0.12	M/D	4.9±0.05	ND^g^
R8A/D547A	0.40±0.07	0.51±0.03	10.7±0.26	1.57±0.17	M/D	3.9±0.04	ND^g^
R8E/D547E	2.77±0.67	2.88±0.51	3.3±0.23	1.11±0.13	M	24.0±0.23	ND^g^
Y237A	0.36±0.04	0.38±0.02	13.9±0.28	1.79±0.19	D	0.29±0.002	ND^g^
E281A	0.45±0.05	0.39±0.01	12.1±0.19	1.95±0.14	D	0.10±0.001	ND^g^
Y435A	2.33±0.43	1.80±0.21	5.9±0.26	1.08±0.07	M	30.3±0.26	ND^g^
Y435N	2.73±0.45	2.16±0.24	4.0±0.19	1.21±0.08	M	33.8±0.24	19.8±0.19
Y237A/E281A	0.38±0.05	0.82±0.06	13.2±0.41	1.60±0.16	D	0.10±0.001	ND^g^
D273A/R544A	0.35±0.04	0.97±0.08	13.5±0.33	1.49±0.09	D	0.68±0.006	ND^g^

a *K*_m,BAEE_: Michaelis constant for benzoyl-L-arginine ethyl ester (BAEE) as the *in vitro* substrate for PAD4.

b *K*_0.5,Ca_: half-saturation constant for Ca^2+^.

c *k*_cat_: catalytic constant.

d *h*: the Hill coefficient.

e M: monomer; D: dimer.

f *K*_d_: dissociation constant between the monomer and dimer without Ca^2+^; *K*_d,Ca_: dissociation constant between the monomer and dimer with 10 mM Ca^2+^.

g ND: not determined.

The mutant with a single substitution of Arg8 with a histidine (R8H) did not dissociate into monomers, and the *K*_d_ value of this mutant was 0.47 µM (Figure 2E); however, substitution of Arg8 with Ala, Lys or Gln caused the dimeric enzyme to dissociate (Figure 3, B, C and D, respectively). The *K*_d_ values for the R8A, R8K and R8Q single mutants were 9.3 µM, 10.2 µM and 15.7 µM, respectively. Asp547, the binding partner of Arg8, was also mutated into Ala, Glu and Asn. The *K*_d_ values for D547A, D547E and D547N mutant enzymes were 6.4 µM, 11.2 µM and 4.9 µM, respectively (Figures 3, E, F and G, respectively). These results suggest that the electrostatic interactions between Arg8 (subunit A) and Asp547 (subunit B) at the dimer interface are important for stabilization of the dimer; however, removal of the ion pair formed by Arg8 and Asp547 did not cause the enzyme to completely dissociate into monomers. The *K*_d_ value of the R8A/D547A double mutant was 3.9 µM (Figure 3H), suggesting that additional factors are involved in PAD4 dimer formation.

Mutation of Arg8 to leucine and glutamate caused the enzyme to predominantly dissociate into monomers, with *K*_d_ values of 16.8 µM for R8L and 45.6 µM for R8E (Figure 4, B and C). The R8E/D547E double mutant was also monomeric (Figure 4D) and had a *K*_d_ value of 24 µM, which also suggests that the electrostatic interactions between Arg8 and Asp547 at the dimer interface are significant.

The Y435A and Y435N mutants were also monomeric (Figure 4, E and F, respectively) and had *K*_d_ values of 30.3 µM and 33.8 µM, respectively. Tyr435 hydrogen bonds to Tyr237 and Glu281 (Figure 1C). In the Y237A/E281A double mutant, the Tyr435-mediated hydrogen-bonding network was not present. The Y237A/E281A double mutant formed a dimer with a similar *K*_d_ value to the WT, which suggests that the monomeric Y435A and Y435N enzymes did not form dimers due to the abolishment of the hydrogen-bonding network but because they lacked the hydrophobicity or geometry related to Tyr435.

**Figure 5:**
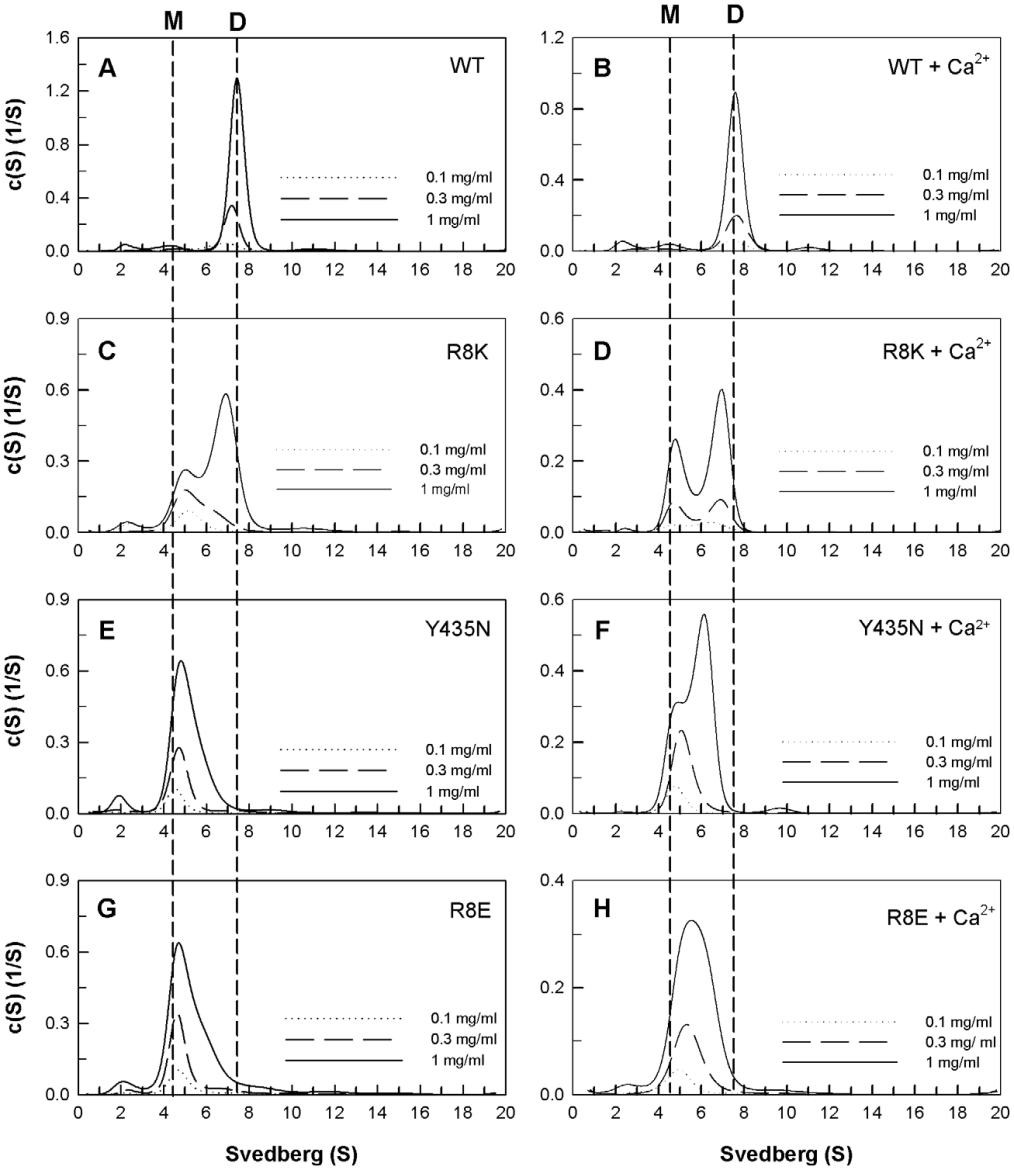
Continuous sedimentation coefficient distributions of the WT PAD4 and dimer interface mutants in the presence of calcium ions. The enzyme concentrations were 0.1, 0.3 and 1.0 mg/mL in 50 mM Tris-HCl and 250 mM NaCl (pH 7.6) at 20°C. The concentration of the calcium ion was 10 mM. (**A**) WT; (**B**) WT with Ca^2+^; (**C**) R8K mutant; (**D**) R8K with Ca^2+^; (**E**) Y435N mutant; (**F**) Y435N with Ca^2+^; (**G**) R8E mutant; (**H**) R8E with Ca^2+^.

We also examined the effect of Ca^2+^ on the quaternary structure of the WT and mutant PAD4 enzymes (Figure 5). Generally, the monomer-dimer equilibrium of PAD4 is not influenced by Ca^2+^. The dissociation constants between the dimeric and monomeric forms of WT and interface mutant PAD4 enzymes in the presence of Ca^2+^ (*K*_d,Ca_) are listed in Table 1. For the dimeric WT enzyme, the *K*_d,Ca_ was 0.51 µM, which is similar to the value in the absence of Ca^2+^ (0.45 µM). For the R8K mutant, which existed in a monomer-dimer equilibrium, the *K*_d,Ca_ was 7.8 µM, which is slightly lower than the *K*_d_ without Ca^2+^ (10.2 µM; Table 1); however, the monomer peak of the R8K mutant did not shift to form more dimers (Figure 5, C and D). Similar results were observed with the Y435N and R8E mutant enzymes. The *K*_d,Ca_ of Y435N was 19.8 µM, which is lower than the value without Ca^2+^ (33.8 µM). For R8E, the *K*_d,Ca_ was 22.3 µM, which is lower than the value without Ca^2+^ (45.6 µM). Although the *K*_d,Ca_ values of these two mutant enzymes were lower than the values without Ca^2+^, the monomeric Y435N and R8E enzymes did not significantly form dimers (Figure 5, E–H). Therefore, the effect of Ca^2+^ on these mutant enzymes may contribute to the conformational stability of the monomeric structure but not to dimer formation.

### Kinetic properties of WT and interface mutant PAD4

For the dimeric PAD4 enzyme, the active site of each monomer is independent and located far away from the dimer interface. Based on this, we assumed that the changes in the enzyme kinetics of these mutant enzymes were more significantly affected by dissociation of the enzyme than by mutation of a specific residue.

The kinetic parameters of the WT and mutant PAD4s are shown in Table 1. The PAD4 enzyme binds Ca^2+^ ions cooperatively. The initial velocities of PAD4, which were measured at various concentrations of Ca^2+^, exhibited sigmoidal kinetics. Table 1 summarizes the results obtained by fitting these sigmoidal curves to the Hill equation. The half-saturation constant for Ca^2+^ (*K*_0.5,Ca_) and the degree of cooperativity of calcium binding (*h*) were estimated.

The Michaelis constant of *in vitro* substrate BAEE (*K*_m,BAEE_) and *K*_0.5,Ca_ values of the PAD4 interface mutant were roughly similar to those of the WT enzyme, indicating that the active site was not significantly perturbed by the mutations; however, the *k*_cat_ values of some of the interface mutants were less than that of the WT enzyme. For dimeric WT PAD4, the *k*_cat_ value was about 13.4 s^−1^. For the R8H, Y237A, Y281A, Y237A/Y281A and D273A/R544A mutants, which existed predominantly as dimers, the *k*_cat_ values were almost the same as that of the WT enzyme.

For the R8A, R8K, R8Q, D547A, D547E, D547N and R8A/D547A mutant enzymes, which existed in monomer-dimer equilibriums, the *k*_cat_ values ranged from 8.9 to 12.2 s^−1^, which represents a slight decrease in the *k*_cat_ and implies that the enzyme activity may be reduced by disrupting the interactions at the subunit interface. For the monomeric PAD4 interface mutants R8L, R8E, R8E/D547E, Y435A and Y435N, the *k*_cat_ values ranged from 3.3 to 7.3 s^−1^. The significant decrease in the *k*_cat_ values of these mutants indicates that the enzymatic activity was reduced due to the dissociation of the dimeric enzyme into monomers. The catalytic activity of monomeric PAD4 was approximately half or less than half of the WT enzyme, which suggests that the monomeric PAD4 enzyme is still active but less active than the dimeric enzyme. In addition, the nine-fold increase in the *K*_m,BAEE_ and *K*_0.5,Ca_ values and the greater than three-fold decrease in the *k*_cat_ values for the R8E/D547E and Y435N mutants may have partially resulted from the instability of the monomeric form of the enzyme, suggesting that dimer formation is advantageous for PAD4 stability.

The *h* value for WT PAD4 was greater than two; however, the *h* values of the interface mutants were significantly reduced (Table 1). The dimeric interface mutant enzymes (Figure 2) had lower *h* values of around 1.5 to 2.0, which indicates that calcium-binding cooperativity is very sensitive to changes in the dimer interface, even though the dimeric structure was maintained. The mutant enzymes that showed a monomer-dimer equilibrium (Figure 3) had *h* values of 1.3 and 1.6, which also indicates that cooperativity was partially lost for these mutant enzymes when the dimer interface was disrupted. For the monomeric interface mutant enzymes (Figure 4), the *h* value was reduced to 1, indicating that the calcium-binding cooperativity for these mutant enzymes was totally lost. The PAD4 monomers were less active and more non-cooperative enzymes.

## Discussion

PAD4 is a homodimer with a fully functional active site in each subunit. The correlation between the catalytic efficiency and dimer formation of this enzyme is unknown. This paper establishes the significance of the dimeric structure of PAD4 for catalysis, regulation and stability.

### Enzyme regulation and subunit-subunit interactions of PAD4

**Figure 6:**
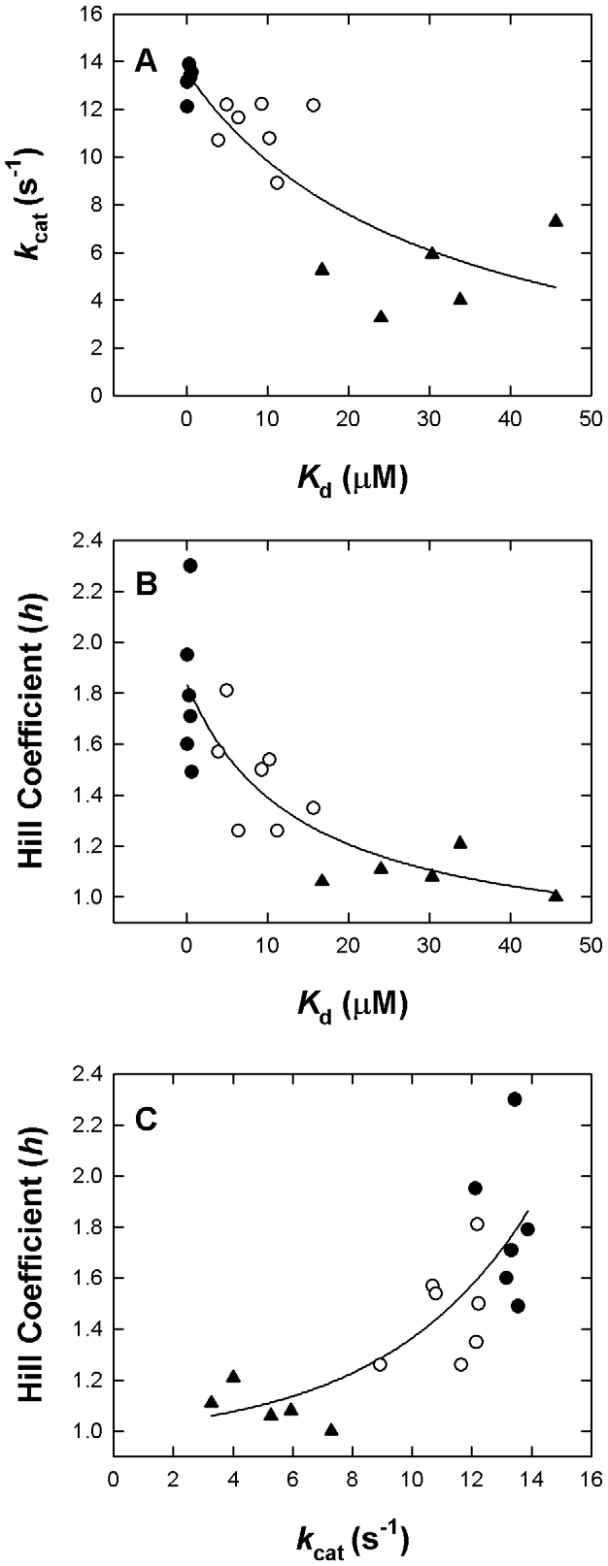
Correlation plots for the dissociation constant (*K*_d_), Hill coefficient (*h*), and catalytic constant (*k*_cat_). The data points were derived from Table 1. Closed circle: dimeric PAD4 (WT, R8H, Y237A, E281A, Y237A/E281A, and D273A/R544A); open circle: PAD4 in dimer-monomer equlibrium (R8A, R8K, R8Q, D547A, D547E, D547N, and R8A/D547A); closed triangle: monomeric PAD4 (R8E, R8L, R8E/D547E, Y435A, and Y435N). **Panel A:** *K*_d_ versus *h*. **Panel B:** *K*_d_ versus *k*_cat_. **Panel C:** *k*_cat_ versus *h*.

The analytical ultracentrifugation and enzyme kinetics analyses clearly revealed that disruption of the dimer interfaces of PAD4 causes the mutants to be less active than the WT protein. Figure 6 shows the correlation between the *K*_d_ and *k*_cat_ values, the *K*_d_ and *h* values and the *k*_cat_ and *h* values. The *k*_cat_ values of these interface mutants decreased with increasing *K*_d_ values (Figure 6A), suggesting that the dissociation of dimers into monomers considerably influences the activity of the enzyme. Furthermore, the *h* values of these interface mutants also decreased with increasing *K*_d_ values, and the *K*_d_ value was closest to 1 when the enzyme dissociated into monomers (Figure 6B), suggesting that the organization at the dimer interface is critical for proper calcium-binding cooperativity. It is not surprising that the increased *k*_cat_ values of these interface mutants corresponded to greater *h* values (Figure 6C). We suggest that the dimer is the fully functional form of human PAD4 because dimer formation correlated with catalytic activity and cooperativity.

### Ionic and hydrophobic interactions stabilize the dimer interface of PAD4

Size distribution analyses demonstrated that the electrostatic interaction between Arg8 and Asp547 and the Tyr435-mediated interactions determine the dimer formation of PAD4. In contrast, the ionic interaction between Asp273 and Arg544 did not seem to be the determinant for PAD4 dimer formation.

Our data also suggested that steric effects are important for the subunit-subunit organization of the PAD4 dimer. The R8H enzyme existed as a dimer while the R8K enzyme was in a monomer-dimer equilibrium (Figures 2 and 3). Similarly, D547E formed a greater proportion of monomers than D547A and D547N (Figure 3). The R8K and D547E enzymes cannot form salt bridges at the dimer interface of the enzyme as well as WT. The improper geometry of the mutant enzyme causes it to dissociate even though the charges of its side chains are preserved.

### The dimer interface residues of peptidylarginine deiminase isoforms

**Figure 7:**
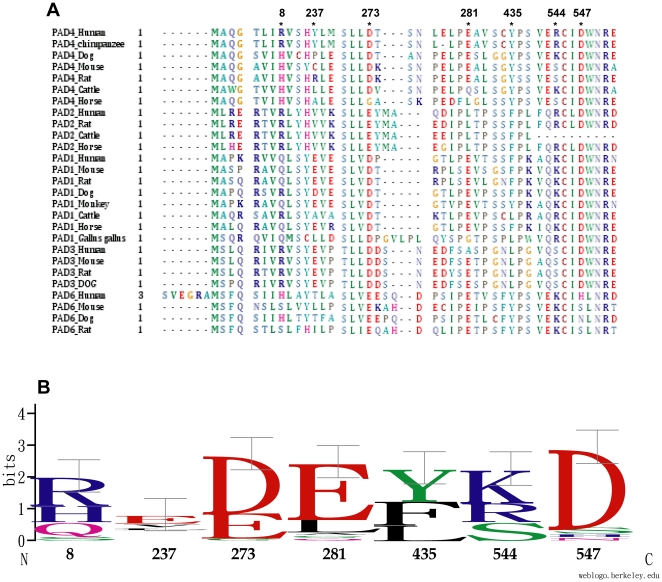
Multiple sequence alignments of the PAD family. The amino acid sequences of PADs were identified using the BLAST [48], and the alignments were generated with ClustalW [49]. **Panel A**: Multiple sequence alignments of 27 isoforms of peptidylarginine deiminase. This figure was generated using the BioEdit sequence alignment editor [50]. **Panel B**: Sequence conservation with error bars for the dimer interface residues. This figure shows the frequency of conservation for the respective amino acid residues at a given position [51].

Figure 7 displays a multiple sequence alignment of the PAD isoforms. Asp547 is highly conserved among the PAD enzymes. Arg8 has a conservative substitution for His, and residue 435 is conserved as a hydrophobic amino acid (Figure 7B). For the PAD4 enzymes, residue 8 is Arg or His, residue 547 is Asp and residue 435 is Tyr. We hypothesize that the PAD4 enzyme in different species should be dimers because the human R8H enzyme is as stable as a dimer as the WT protein. Residues 8 and 547 in PAD2 and PAD3 are Arg and Asp, respectively. Residue 435 is Phe in PAD2 and Leu in PAD3, both of which are hydrophobic residues similar to Tyr. We postulate that Tyr435 in human PAD4 is involved in the hydrophobic interactions in the dimer interface of the enzyme and that Phe435 in PAD2 and Leu in PAD3 should have similar hydrophobic effects in the interface of the enzyme. Therefore, we propose that the PAD2 and PAD3 enzymes may also be stable dimers. For some PAD1 enzymes in different species, residue 8 is Gln, and the other two residues are conserved. It is possible that the PAD1 enzymes might be unstable dimers because the human R8Q enzyme displayed a monomer-dimer equilibrium. For PAD6, residue 435 is Tyr, but the ion pair between residues 8 and 547 is not conserved. Our data indicate that the human R8A/D547A enzyme is in equilibrium between the monomeric and dimeric forms, with a nine-fold larger *K*_d_ value than the WT protein. The PAD6 enzyme, in which Tyr435 is conserved, may sufficiently dimerize; however, the quaternary structures of the PAD isoforms need further investigation in the future.

### Conclusion

In summary, we suggest that the dimerization of PAD4 is essential for full enzyme activity and calcium-binding cooperativity. Monomeric PAD4 maintains 25–50% enzyme activity compared to the dimeric enzyme, suggesting that the PAD4 monomer is still active. Although PAD4 is the only isoform of the PAD family for which a crystal structure has been determined [42], our data suggest that other PAD isoforms may also exist as dimers with different association affinities. In addition, excess PAD4 expression seems to be associated with rheumatoid arthritis. Our data may provide a rationale for the development of PAD-inhibiting drugs by disrupting the dimerization of PAD4 to decrease its activity.

## Materials and Methods

### Site-Directed Mutagenesis

Site-directed mutagenesis was performed using the QuikChange™ mutagenesis kit (Stratagene, USA) to create the mutant human PAD4 constructs. The human PAD4 DNA was purchased from RZPD Deutsches Ressourcenzentrum für Genomforschung. The purified PAD4 DNA was used as template, and primers with the desired codon changes were used for PCR (polymerase chain reaction) with high fidelity *Pfu* DNA polymerase. The primers with the desired mutations ranged from 25 to 45 bases in length, which were required for specific binding to the template DNA. After 16–18 temperature cycles, mutated plasmids that included staggered nicks were produced. The PCR products were treated with DpnI to digest the wild-type human PAD4 templates, and the nicked DNA with the desired mutations was transformed into the XL-1 strain of *Escherichia coli*. The sequences of the mutants were verified by dideoxy-based sequencing.

### Expression and Purification of Recombinant PAD4

PAD4 was sub-cloned into the pQE30 vector, which carries an N-terminal 6His tag. This ampicillin-resistant vector was transformed into the JM109 strain of *Escherichia coli*. PAD4 expression was induced with 1.0 mM isopropyl-1-thio-β-D-galactoside (IPTG) at 25°C overnight. The over-expressed enzyme was purified using Ni-NTA Sepharose (Sigma). The lysate-Ni-NTA mixture was washed in a stepwise fashion (5, 10 and 20 mM imidazole in 500 mM sodium chloride, 2.5 mM dithiothreitol and 30 mM Tris-HCl, pH 7.6) to remove unwanted proteins, and then PAD4 was eluted with elution buffer (80 mM imidazole, 500 mM sodium chloride, 2.5 mM dithiothreitol and 30 mM Tris-HCl, pH 7.6). The purified enzyme was then buffer exchanged into 50 mM Tris-HCl (pH 7.6), 500 mM NaCl and 2 mM β-mercaptoethanol and concentrated using a centrifugal filter device (Amicon Ultra-15, Millipore) with a molecular weight cutoff of 30 kDa. The enzyme purity was verified by resolving the eluted protein with sodium dodecyl sulfate-polyacrylamide gel electrophoresis (SDS-PAGE) and staining with Coomassie, and protein concentrations were approximately calculated with the Bradford method [43].

### Enzyme assay of PAD4 and analysis of kinetic data

A protocol for the continuous measurement of PAD4 enzyme activity, which is coupled to a glutamate dehydrogenase-catalyzed reaction, has been reported [44]. The reaction mixture for the spectrophotometric assay of PAD4 activity contained 10 mM benzoyl-L-arginine ethyl ester (BAEE) as the *in vitro* substrate for PAD4, 10 mM CaCl_2_, 2.5 mM dithiothreitol, 8.5 mM α-ketoglutarate (α-KG), 0.22 mM NADH and 8.4 U of glutamate dehydrogenase (GDH) in 100 mM Tris-HCl (pH 7.5) in a total volume of 1 ml at 25°C. The reaction was initiated by the addition of the appropriate amount of enzyme to the reaction mixture, and the decrease in absorbance at 340 nm was continuously monitored using a Perkin-Elmer Lamba-25 spectrophotometer. An enzyme unit is defined as the amount of enzyme that catalyzed the decomposition of 1 µmol of NADH per min. An extinction coefficient of 6220 M^−1^ for NADH was employed in the calculations. The apparent Michaelis constants were determined for the BAEE substrate by varying its concentration near its *K*_m_ value with invariable and saturated concentrations of the other constituents.

Furthermore, the *k*_cat_ values of PAD4 were calculated with the following equation:
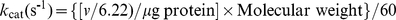



where *v* represents ▵A_340_/min, 6.22 is the millimolar absorption coefficient of NAD(P)H, 148,000 is the molecular weight of the human PAD4 dimer and 60 is the number of seconds per minute.

The sigmoidal curves of [Ca^2+^] versus the initial velocities were input into the Hill equation, and the data were further analyzed to calculate the *K*_0.5,Ca_ value, which is the calcium concentration at half-maximal velocity, and the Hill coefficient (*h*), which were utilized to evaluate the degree of cooperativity using the following equation:
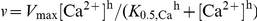



Graphical analysis of the data was performed with the Sigma Plot 8.0 program (Jandel, San Rafael, CA).

### Analytical ultracentrifugation

The quaternary structure of PAD4 was analyzed with an Optima XL-A Analytical Ultracentrifuge (Beckman, USA). The concentrations of the PAD4 enzyme in sedimentation velocity (SV) experiments were fixed at 0.1, 0.3, and 1 mg/ml. The sample cell was loaded with 380 µl of sample, and the reference cell was loaded with 400 µl of 50 mM Tris-HCl and 250 mM NaCl (pH 7.6). After the protein samples were loaded, the cells were transferred into an An-50 Ti analytical rotor. The enzyme was detected at an absorbance of 280 nm in continuous mode with high-speed centrifugation (42000 rpm) for 4 hours at 20°C, with a time interval of 480 seconds and a step size of 0.002 cm. Numerous scans of the sedimentation velocity data were collected and analyzed globally with the SEDFIT 9.4c software [45]. All size distributions were determined with a confidence level of p = 0.95, a best-fitted average anhydrous frictional ratio (*f/f_0_*), and a resolution of 200 sedimentation coefficients between 0.1 and 20.0 S.

### Self-association of the PAD4 enzyme

The dissociation constant (*K*_d_) of PAD4 was analyzed using the SEDPHAT program with a monomer-dimer equilibrium model [45], [46]. The sedimentation velocity data, which were collected with three different enzyme concentrations, were globally fitted with SEDPHAT, and the partial, specific volume of the enzyme, solvent density, and viscosity were calculated by the SEDNTERP program [47].
